# Analysis and modification of central carbon metabolism in *Hypsizygus marmoreus* for improving mycelial growth performance and fruiting body yield

**DOI:** 10.3389/fmicb.2023.1233512

**Published:** 2023-07-25

**Authors:** Hui Lin, Pengfei Li, Lu Ma, Shufang Lai, Shujing Sun, Kaihui Hu, Liaoyuan Zhang

**Affiliations:** ^1^Department of Bioengineering, College of Life Sciences, Fujian Agriculture and Forestry University, Fuzhou, Fujian, China; ^2^Institute of Edible Fungi, Fujian Academy of Agricultural Sciences, Fuzhou, Fujian, China; ^3^Fujian Edible Fungus Technology Promotion General Station, Fuzhou, Fujian, China

**Keywords:** *Hypsizygus marmoreus*, harvest period, central carbon metabolism (CCM), pentose phosphate pathway (PPP), glucose-6-phosphate dehydrogenase (G6PDH)

## Abstract

*Hypsizygus marmoreus* is one of the main industrially cultivated varieties of edible fungi, with a delicious taste and high nutritional value. However, the long harvest period of 130–150 days greatly limits its large-scale expansion. This study aimed to investigate the effects of central carbon metabolism (CCM) on the mycelial growth performance and fruiting body formation of *H. marmoreus*. Nine edible fungi with different harvest periods were collected and used to evaluate their intracellular carbon metabolic differences in the CCM, which revealed that the imbalanced distribution of intracellular carbon metabolic levels in the CCM of *H. marmoreus* might be one of the key factors resulting in a slow mycelial growth rate and a long harvest period. Further analysis by three strategies, including metabolomics, adaptation of different carbon sources, and chemical interference, confirmed that low carbon flux into the pentose phosphate pathway (PPP) limited the supply of raw materials, reduced power, and thus influenced the mycelial growth of *H. marmoreus*. Furthermore, four transformants with increased expression levels of glucose-6-phosphate dehydrogenase (G6PDH), a key rate-limiting enzyme in the PPP of *H. marmoreus*, were developed and showed more extracellular soluble protein secretion and higher sugar assimilation rates, as well as improved mycelial growth rates in bottle substrate mixtures. Finally, cultivation experiments indicated that the maturation periods of the fruiting body with ~4–5 days in advance and the maximum fruiting body yield of 574.8 g per bag with an increase of 7.4% were achieved by improving the G6PDH expression level of the PPP in *H. marmoreus*. This study showed that CCM played an important role in the mycelial growth and development of *H. marmoreus*, which provided new insights for future advancements in cultivating and breeding edible fungi.

## Introduction

As one of the most important cash crops, edible fungi are considered a healthy food due to their high protein content, carbohydrates, fiber, vitamins, minerals, and low-fat content (Kumla et al., 2020). Most of the cultivated edible fungi are classified as saprophytic fungi, capable of breaking down lignocellulosic materials to obtain nutrients by secreting a wide range of enzymes, such as cellulases, hemicellulases, and other carbohydrate-active enzymes (CAZymes) (Cai et al., 2021). The breakdown of lignocellulosic materials, which consist of cellulose, xylan, and pectin, mainly results in the release of monosaccharides such as glucose, xylose, and arabinose (Horn et al., 2012; Dos Santos et al., 2018), which are then transported into the fungal cell for assimilation with the help of sugar transporters (Sloothaak et al., 2016). Therefore, the lignocellulosic materials utilized by edible fungi can be divided into two sections, including extracellular degradation and intracellular metabolism, which contribute to the availability of nutrients and energy for the growth and development of edible fungi (Mattila et al., 2020). To improve the edible fungi growth rate and fruiting body yield, many previous studies mainly focused on increasing the supply of nutrients by overexpressing the CAZyme gene (Zhang et al., 2015; Yan et al., 2021), and the research found that PoV mycovirus affects the spawn growth and fruiting body formation of *Pleurotus ostreatus* via the decreased expression of extracellular enzymes (Song et al., 2020). These results suggested that the overexpression of extracellular enzymes could accelerate the decomposition of lignocellulosic materials and thus provide more available nutrients for the growth and development of edible fungi. However, few studies regarding edible fungi paid attention to the relationship between intracellular carbon metabolism and the growth and development of edible fungi. Intracellular monosaccharide assimilation could contribute to adequate materials and energy for the growth and development of edible fungi. Therefore, intracellular carbon metabolic level might play a critical role in the growth rate and fruiting body yield of edible fungi.

The core components of intracellular carbon metabolism, including the Embden Meyerhof pathway (EMP), pentose phosphate pathway (PPP), and tricarboxylic acid cycle (TCA), are collectively referred to as central carbon metabolism (CCM) (Yan Z. Y. et al., 2021), which plays a vital role in providing primary metabolites and energy for the cell. These primary metabolites serve as precursor molecules for DNA, RNA, protein, and other essential cellular components, contributing to cell growth and the maintenance of cellular functions. Furthermore, they also act as a source for the production of secondary metabolites and extracellular enzymes. A previous study also showed that the DNA, RNA, and protein contents of the cell appeared to have a positive relationship with the cell growth rate, and faster-growing cells contained a greater number of these macromolecules (Schaechter et al., 1958). However, the effect of CCM levels and their carbon flux distribution on the growth and development of edible fungi was rarely reported. In addition, intracellular carbon metabolism showed a noticeable impact on the expression and secretion of CAZyme in filamentous fungi by carbon catabolite repression (CCR) (Adnan et al., 2018). The Cys_2_His_2_-type transcription factor CreA/CREI involved in glucose regression had been cloned from numerous filamentous fungi and exhibited the ability to regulate cellulase expression for improving lignocellulose utilization (Wu et al., 2020). D-xylose utilization could result in increasing hemicellulase expression levels in filamentous fungi mediated by XlnR/XYR1 (Xia et al., 2022). These results demonstrated the close connection between extracellular polysaccharide decomposition and CCM (Delgado-Jarana et al., 2003; Matsushika et al., 2012).

*Hypsizygus marmoreus* (Peck) Bigelow belongs to the Agaricales or Basidiomycetes. As one of the most economically important edible fungi, it is widely cultured in China, South Korea, and Japan (Wu et al., 2019). It has great market potential due to its unique flavor and crunchy texture. *H. marmoreus* is also an important medicinal mushroom that contains abundant biologically active compounds, including novel proteins (Shoji et al., 2000), polysaccharides (Yan et al., 2014), and terpenoids (Yoshino et al., 2008). As a result, the cultivation scale of *H. marmoreus* via the bag or bottle substrate cultivation method increased rapidly in different regions of the world, especially in China. The cultivation process could be divided into five typical stages: inoculation, mycelium growth, after-ripening, primordium, and fruiting body stages. This complete process from inoculation to fruiting body harvest was called the one-harvest period, and the harvest period of *H. marmoreus* needed 140–150 days due to ~110–120 days of mycelium growth and after-ripening stages (Zhang et al., 2021). The long harvest period resulted in high production costs, low factory availability efficiency, and poor resistance. Curiously, the harvest period of different edible fungi species varied greatly, such as *Volvaria volvacea, P. ostreatus*, and *Pleurotus pulmonarius*, with fast mycelial growth rates that only needed 30–50 days to complete one harvest period (Zervakis et al., 2001). The important question is whether the harvest period of edible fungi species is related to intracellular carbon metabolic levels or not. Therefore, in this study, nine representative edible fungi species with different harvest periods were collected from a commercial mushroom factory and identified by ITS sequencing. Subsequently, mycelial growth performance, glucose assimilation rates, and the activities of rate-limiting enzymes involved in CCM by all the test strains were measured and compared to explore the intracellular carbon flux distribution in *H. marmoreus*, and the obtained results were verified using metabolomics, adaptation analysis of various carbon sources, and chemical interference methods. The results showed noticeable differences in central carbon metabolic levels in edible fungi species with different harvest periods. The imbalanced distribution of central carbon metabolism and low metabolic level of the PPP in *H. marmoreus* was a key factor resulting in a slow mycelial growth rate. Ultimately, the adjustment of intracellular carbon metabolic flux by enhancing the expression level of the *g6pd* gene encoding glucose-6-phosphate dehydrogenase (G6PDH), a key rate-limiting enzyme of PPP in *H. marmoreus*, efficiently improved its mycelial growth rate and fruiting body yield. This study attempted to determine and analyze the effects of CCM on mycelial growth and the development of edible fungi for the first time. These results provided new insights for the breeding of edible fungi in the future.

## Materials and methods

### Strains and isolation

Nine edible fungi fruiting bodies of *V. volvacea, P. ostreatus, P. pulmonarius, Pleurotus eryngii, Agrocybe cylindracea, Flammulina velutipes, H. marmoreus* (BY), *H. marmoreus* (19C), and *Lentinula edodes* were collected from a commercial mushroom farm in Fujian Province, China (Supplementary Figure 1). Tissue masses were immersed in a 75% ethanol solution for 1 min, followed by a 4% sodium hypochlorite solution for 3 min. Then, tissue masses were rinsed with sterile distilled water 3–4 times to remove the residual ethanol and sodium hypochlorite. The sterilized pileus surface parts were cut into some tissue masses with a sterile scalpel and inoculated onto potato dextrose agar (PDA) medium with 50 mg/L kanamycin (Kan^R^). Plates were incubated at 25°C, and further purification through twice-hypha tip isolation was performed after hyphae grew out at the edge of the tissue wound.

### Mycelial growth rate and biomass assay

Nine edible fungi were inoculated on plates containing solid enrichment PDA medium (PDA medium with 2 g peptone, 2 g yeast powder, 3 g KH_2_PO_4_, 1.5 g MgSO_4_, and 0.1 g Vitamin B_1_) in darkness at 25°C until the colony diameters filled the medium surface. One piece of mycelial blocks was aseptically picked and placed on the center of a solid substrate media (SM) plate (10 g/L filter paper, 10 g/L rice husk, 5 g/L bran, 2 g/L CMC-Na, 0.5 g/L MgSO_4_, 0.5 g/L KH_2_PO_4_, 1 g/L K_2_HPO_4_, 20 g/L power agar) and a solid glucose media (GM) plate [20 g/L glucose, 2 g/L peptone, 2 g/L (NH_4_)_2_SO_4_, 0.5 g/L MgSO_4_, 0.5 g/L KH_2_PO_4_, 1 g/L K_2_HPO_4_, 20 g/L power agar] cultured in the dark at 25°C. The center of the mycelium block was chosen as the cross point to draw a vertical line. The growth length of mycelium was recorded every day until the colony diameters filled the plate, and then the average mycelium growth rate was calculated.

For biomass determination, nine edible fungi were cultured in a liquid substrate medium and a liquid glucose medium, respectively. For the liquid substrate medium, a piece of aseptically preprocessed filter paper was placed on the surface media to avoid substrate adsorption on the hypha. The mycelium biomass from two kinds of medium was collected via centrifugation after cultivation for 7 days and washed the mycelium twice with deionized water. After excess water on the hypha was removed using filter paper, the biomass concentration as wet weight was determined. The average wet weight was calculated and recorded.

### Enzyme activity and glucose concentration assays

All the mycelia were cultured in a liquid glucose medium and subsequently collected by centrifugation. The obtained mycelia were rinsed with precooled phosphate buffer (50 mmol/L, pH 6.5) three times and stored at −80°C after removing the residual buffer from the hypha with filter paper. The crude extracts of intracellular enzymes and glucose were obtained using liquid nitrogen grinding and ultrasonic treatment. In brief, the frozen mycelia were transferred into a sterile, pre-cooled mortar and ground to a powder under liquid nitrogen with a pestle. Subsequently, these powders were transferred to a 10-ml centrifuge tube and resuspended in a 10-fold (w/v) extraction buffer. The extraction buffer consisted of 10% (v/v) glycerol, 0.25% (w/v) BSA, 0.1% (v/v) Triton X-100, 10 mM MgCl_2_, 1 mM EDTA, 1 mM EGTA, 1 mM benzamidine, 1 mM 6-amino capronic acid, 1 mM phenylmethylsulfonyl fluoride (added before use), 10 mM leupeptin, and 1 mM DTT, 50 mM HEPES-KOH buffer, pH 7.5. The resuspended mycelia were ultra-sonicated by a JY92-IIN Ultrasonic cell crusher with 40% power for 3 s (9 s intervals) until this solution was clear. The cell debris was discarded by centrifugation at 10,000 rpm for 10 min at 4°C, and the supernatant was laid on ice for intracellular enzymes and glucose assays.

Hexokinase (HK, EC: 2.7.1.1) activity was determined as described below: the reaction mixture contained 50 mM triethanolamine buffer (pH 7.9), 20 μl crude enzyme extract, 5 mM MgCl_2_, 5 mM ATP, 1 mM NADP^+^ and 2 units/ml glucose-6-phosphate dehydrogenase. The addition of 20 mM glucose started the reaction, and the absorbance changes at 340 nm were spectrophotometrically recorded. The reaction without glucose-6-phosphate dehydrogenase was used as the blank.

Phosphofructokinase (PFK, EC: 2.7.1.11) activity was determined as described below: the reaction mixture contained 50 mM HEPES-KOH buffer (pH 7.8), 5 mM MgCl_2_, 1 mM DTE, 0.2 mM NADH, 1 mM ATP, 1 mM fructose 6-phosphate, and 10 units/ml aldolase, 5 units/ml triose-phosphate isomerase, and 5 units/ml glycerol 3-phosphate dehydrogenase. The reaction was started by the addition of 25 μl of the crude enzyme extract and spectrophotometrically monitored at 340 nm.

Pyruvate kinase (PYK, EC: 2.7.1.40) activity was determined as described below: the reaction mixtures contained 50 mM (MOPS)-KOH buffer (pH 7.0), 5% polyethylene glycol 8,000, 50 mM KCl, 15 mM MgCl_2_, 1 mM DTT, 2 mM PEP, 1 mM ADP, 0.2 mM NADH, and 2 units/ml rabbit muscle lactate dehydrogenase. The reaction was started with the addition of 20 μl of crude enzyme extract and spectrophotometrically monitored at 340 nm.

Citrate synthase (CS, EC: 2.3.3.1) activity was determined as described below: the reaction mixture contained 50 mM Tris-HCl buffer, pH 8.0, 100 μl crude enzyme extract, 0.1 mM 2-nitrobenzoic acid, and 0.2 mM acetyl-CoA. The reaction was started by adding 0.6 mM oxaloacetate and spectrophotometrically monitored at 412 nm. The extinction coefficient used was 14,150 M^−1^ cm^−1^.

NADP-dependent isocitrate dehydrogenase (IDH, EC: 1.1.1.42) activity was determined as described below: the reaction mixture contained 50 mm HEPES buffer (pH 7.5), 25 μl crude enzyme extract, 2 mM MgCl_2_, and 1.2 mM NADP^+^. The reaction was initiated by the addition of 20 mM isocitrate and spectrophotometrically monitored at 340 nm. The NAD-dependent isocitrate dehydrogenase (IDH, EC: 1.1.1.41) activity assay was performed by a method similar to NADP-dependent isocitrate dehydrogenase, and only the NADP^+^ coenzyme was replaced with NAD^+^. The sum of activities from both IDH enzymes was used to calculate.

2-Ketoglutarate dehydrogenase (KGDH, EC: 1.2.4.2) activity was determined as described below: the reaction mixture contained 50 mM phosphate buffer (pH 8.0), 2.5 mM NAD^+^, 0.2 mM TPP, 1 mM MgCl_2_, 0.13 mM CoA, 2.6 mM cysteine, and 2.0 mM sodium 2-oxoglutarate. The reaction was started by the addition of 20 μl of crude enzyme extract and spectrophotometrically monitored at 340 nm.

Glucose-6-phosphate dehydrogenase (G6PDH, EC: 1.1.1.49) activity was determined as described below: the reaction mixtures contained 50 mM Tris-HCl, pH 8.0, 5 mM MgCl_2_, 1 mM NADP^+^, and 2.5 mM glucose-6-phosphate. The reaction was started by the addition of 20 μl of crude enzyme extract and spectrophotometrically monitored at 340 nm.

Intracellular glucose concentration and extracellular glucose consumption were assayed using a glucose assay kit (Rong Sheng Biotech, Shanghai).

### Intracellular nucleoside assay

The mycelia of nine edible fungi cultured in a liquid glucose medium were collected via centrifugation. Assays of intracellular nucleoside concentrations were performed using a slightly modified procedure as described in a previous study (Vrabl et al., 2017). The mycelia were rinsed with precooled HEPES buffer (70 mM, pH 6.5) with aqueous 60% methanol (v/v) (−40°C) three times, and the residual buffer on the hypha was removed by filter paper after centrifugation. The wet mycelia were resuspended in 10-fold (w/v) HEPES buffer (70 mM, pH 5.2) with aqueous 75% ethanol (v/v) and incubated at 90°C for 10 min. Subsequently, the suspension was cooled on ice for 10 min, and the cell debris was removed by centrifugation at 10,000 rpm and 4°C for 20 min. Finally, the supernatant was dried with a vacuum drier for further use. Prior to analysis, the lyophilized samples were resuspended in distilled water and filtered through a 0.22 μm filter membrane (Millipore, Germany). The ATP, ADP, NADH, NAD^+^, NADPH, and NADP^+^ in the prepared samples were analyzed and quantified using HPLC (Agilent 1100, USA) equipped with a DAD detector by the following procedure. Gradient elution was conducted using a mobile phase with 95% phosphate buffer (0.1 M, pH 7.0) and 5% acetonitrile on an SB-aq C18 reverse-phase column at 20°C. The flow rate was controlled at 0.8 ml/min, with a total run time of 35 min. The signal was monitored at 254 nm, and the concentrations of ATP, ADP, NADH, NAD^+^, NADPH, and NADP^+^ were calculated according to the strand curve generated by the corresponding standard samples.

### Metabolomics analysis

The mycelia of *H. marmoreus* (BY) and *H. marmoreus* (19C) were cultured and collected for metabolomics analysis, and the strains of *V. volvacea, F. velutipes*, and *L. edodes*, which belong to S-, M-, and L-types, respectively, were used as controls. These collected mycelia were rinsed with precooled, sterile, distilled water two times. After removing residual water from the hypha with sterile filter paper, the mycelia were quickly frozen by liquid nitrogen and stored at −80°C. The samples were analyzed via metabolomics by a commercial company (Metware Biotech, Wuhan). Metabolomics analysis is based on widely targeted metabolomics technology. The data acquisition system mainly includes ultra-performance liquid chromatography and Tandem mass spectrometry.

### Adaptation analysis of various carbon sources

Various carbon sources, including glucose, xylose, ribose, and sodium pyruvate, were used to evaluate the effect of glycometabolism on the mycelial growth rate and phenotype of *H. marmoreus* (BY) and *H. marmoreus* (19C) and the strains of *V. volvacea, F. velutipes*, and *L. edodes*, which belong to S-, M-, and L-types, respectively, were used as the control. The minimal media (per liter) consisted of 8 g Na_2_HPO_4_, 1.5 g KH_2_PO_4_, 0.25 g NaCl, 2.5 g NH_4_Cl, 1 mM MgCl_2_, 0.1 mM CaCl_2_, 0.25 g peptone, and 20 g power agar, plus 2% (w/v) of each carbon source. The experiments were performed in quintuplicate for each carbon source. The diameters of colonies were measured and recorded after 3 days.

### Chemical interference with central carbon metabolism

Seven kinds of different chemical activators or inhibitors were used to interfere with the central carbon metabolism of *H. marmoreus* (BY) and *H. marmoreus* (19C), and their effects on the mycelial growth rate and phenotype of *H. marmoreus* were assayed using glucose media plates that contained one of the following interferent agents: 0.2 mM 6-amino-nicotinamide (6AN) (Almugadam et al., 2018), 0.1 mM dehydroepiandrosterone (DEHA) (Mele et al., 2018), 1 mM valproate (VA) (Debeb et al., 2012a), 50 μM suberoylanilide hydroxamic acid (SAHA) (Debeb et al., 2012b), 2 mM dichloroacetic acid (DCA) (Wu et al., 2018), 10 mM succinate, and 2 mM malonate (Cheng et al., 2017), which were sterilized by 0.22 μm membrane filtration before use. The strains of *V. volvacea, F. velutipes, and L. edodes* belonging to S-, M-, and L-types were used as controls. The experiments were performed in quintuplicate.

### Promoter replacement of the *g6pd* gene in *H. marmoreus*

The homologous recombination fragment for the replacement of the native *g6pd* promoter comprised four segments: *g6pdup, hph*, P_*gpd*_, and *g6pddn*. The *g6pdup* and *g6pddn* fragments represented the upstream homologous sequence (1,119 bp) of the *g6pd* gene and its partial sequence (952 bp) from *H. marmoreus* (BY) and *H. marmoreus* (19C), respectively, which were used to perform homologous recombination. The *hph* fragment contained the hygromycin B resistance gene and terminator sequence derived from the plasmid pCAMBIA1301, and its expression was activated by the native promoter of the *g6pd* gene from the *g6pdup* fragment and used for the initial screening of transformants. In contrast, the P_*gpd*_ fragment contained the native promoter of glyceraldehyde phosphate dehydrogenase (GPD) from *H. marmoreus* (BY) and *H. marmoreus* (19C), which was used to drive overexpression of G6PDH in *H. marmoreus* (BY) and *H. marmoreus* (19C). Four fragments were amplified using the corresponding primers (Supplementary Table 1) and assembled into the cloning vector pUC19 using the ClonExpress MultiS One-Step Cloning Kit. The recombinant plasmid was transformed into *Escherichia coli* DH5α for commercial sequencing. The recombinant plasmid as a template was used to amplify the homologous recombination fragment, which was recovered and used for PEG-mediated transformation, as described in a previous study (Gao et al., 2017). The transformants were screened in the presence of 4 μg/ml hygromycin B and verified by PCR and commercial sequencing.

### Quantitation of the transcriptional levels of g6pd by qRT-PCR

TaqMan qRT-PCR was used to monitor transcriptional levels of the *g6pd* gene in the transformants, and the wild-type strains were used as controls. The qRT-PCR was performed as previously described (McGann et al., 2007). All the qRT-PCR experiments were performed in triplicate.

### Assay of glucose assimilation rate by wild-type strains and transformants

Wild-type strains and transformants were cultured on plates that contained enrichment PDA agar medium in darkness at 25°C until the colony diameters filled the medium surface. Subsequently, the pieces of mycelial blocks were aseptically picked and placed on the center of a liquid glucose media plate, which was cultured in the dark at 25°C. The mycelial growth rate, biomass, and glucose consumption were determined during the culture process.

### Cultivation tests

For the cultivation tests, wild-type strains and transformants were inoculated in liquid spawn medium (200 g potato, 20 g glucose, 3 g KH_2_PO_4_, 1.5 g MgSO_4_, and 5 g Soybean meal per liter) and cultured at 25°C in the dark for 7 days with an agitation rate of 120 rpm. The mycelia were then inoculated into the cultivation medium in bottles and bags. The cultivation medium consisted of cottonseed hull, sawdust, wheat bran, cornmeal, and lime, which were mixed at a ratio of 50:20:25:4:1, and the humidity was adjusted to ~60%. The wet substrates of 1.4 kg in bags and 0.28 kg in bottles were sterilized at 121°C for 150 min. Each bag and bottle were inoculated with liquid spawn and incubated in the dark at 25°C. For bottle cultivation, the mycelial growth rates of wild-type strains and transformants were measured and recorded. Moreover, the extracellular metabolic parameters, including soluble protein, reducing sugar, and the activity of CMCase, FPase, xylanase, and amylase, by four transformants and wild-type strains, were determined as described in previous studies when mycelial filled half of the cultivation medium in a bottle (Dhali et al., 2021). For bag cultivation, the bags were transferred to the fruiting room for fruiting after overgrowing and ripening (110 days). Fruiting bodies were harvested from the substrates in the bags after maturity. The yields of fruiting bodies by wild-type strains and transformants were determined via the weighting method.

## Results and discussion

### The correlation analysis between the harvest period and mycelial growth performance

**Figure 1:**
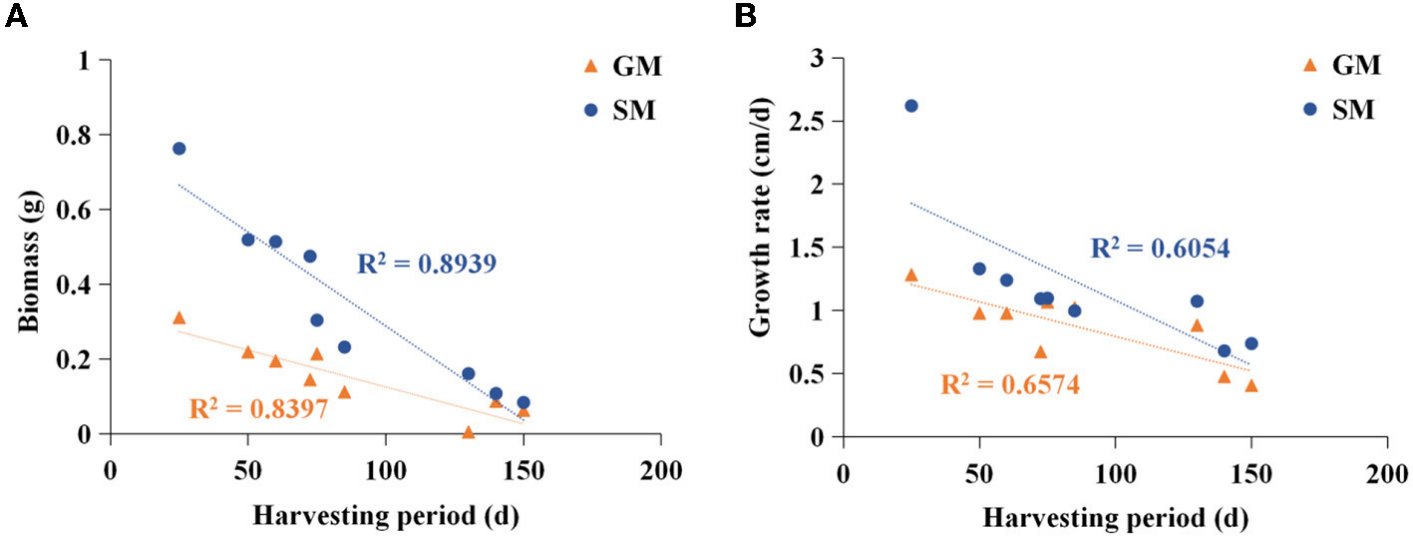
Analysis of correlation between harvesting period and biomass **(A)**/mycelial growth rate **(B)**. SM, substrate media; GM, glucose media.

The edible fungi strains used for mushroom production frequently degenerate during their mycelial subculturing process. The mycelia of degenerated edible fungi appeared slim, fragile and had a slow growth rate, which resulted in low fruiting body yield (Chen et al., 2019; Pérez et al., 2021). Therefore, to ensure the performance reliability of the initial strain, the fruiting bodies of nine commercial edible fungi with different harvest periods were collected from mushroom farms (Supplementary Table 1). According to the harvest periods, these nine strains were divided into three types: long harvest period [L-type, *H. marmoreus* (19C), *H. marmoreus* (BY), and *L. edodes*], medium harvest period (M-type, *A. cylindracea, F. velutipes*, and *P. eryngii*), and short harvest period (S-type, *P. pulmonarius, P. ostreatus*, and *V. volvacea*; Table 1). The tissue and hyphal tip were isolated to obtain pure strains of nine edible fungi. The results of ITS sequencing showed that all the pure strains were successfully obtained from these fruiting bodies. Subsequently, nine pure edible fungi were cultured to analyze their growth performance using solid SM/GM medium and liquid SM/GM medium, respectively. Microscopic morphology showed a slim and fragile mycelial with a minor diameter for the mycelial of M- and L-type strains, while a stout and strong mycelial with a large diameter could be observed in the S-type strains (Supplementary Figure 2). In a previous study, a sharp transition from thick to thin hyphae in response to carbon starvation was observed in *Aspergillus niger*, suggesting that the mycelial morphological characteristic was closely related to the carbon metabolic level (Nitsche et al., 2012). Furthermore, the mycelial growth rates and biomass exhibited significant differences among nine edible fungi (Table 2). The L-type strains of *H. marmoreus* (19C), *H. marmoreus* (BY), and *L. edodes* exhibited the slowest growth rates and least biomass, while the fastest growth rates and most biomass could be achieved by the S-type strains of *P. pulmonarius, P. ostreatus*, and *V. volvacea*, and those of the M-type strains of *A. cylindracea, F. velutipes*, and *P. eryngii* were between the L-type and S-type. These results indicated that the growth performance of nine edible fungi was closely related to their corresponding harvest periods. As shown in Figure 1, the correlation analysis showed that the harvest periods appeared to have a significantly negative correlation with the mycelial growth rates (correlation coefficient squared *R*^2^ of 0.6054 and 0.6574) and biomass (correlation coefficient squared *R*^2^ of 0.8939 and 0.8379). Additionally, the substrate medium with indirect carbon sources of filter paper, bran, and rice husk exhibited a similar trend in mycelial growth rates and biomass to the glucose medium containing a direct carbon source in glucose among nine edible fungi (Table 2), suggesting that intracellular monosaccharide assimilation levels might play an important role in the mycelial growth rates of edible fungi. The above results encouraged us to explore the differences in intracellular monosaccharide metabolism among nine edible fungi.

**Table 1:** The harvest periods of nine edible fungi.

**Strains**	**Harvest period (day)**	**Classification**
*Hypsizygus marmoreus* (19C)	150	Long harvest period (L type)
*Hypsizygus marmoreus* (BY)	140	
*Lentinula edodes*	130–140	
*Agrocybe cylindracea*	80–90	Medium harvest period (M type)
*Flammulina velutipes*	70–80	
*Pleurotus eryngii*	70–75	
*Pleurotus pulmonarius*	55–65	Short harvest period (S type)
*Pleurotus ostreatus*	45–55	
*Volvaria volvacea*	25–30	

**Table 2:** Determination of mycelial growth rates on solid medium and biomass on liquid medium.

**Strains**	**Biomass (g)**		**Average growth rate (cm/day)**	
	**Substrate media (SM)**	**Glucose media (GM)**	**Substrate media (SM)**	**Glucose media (GM)**
*Hypsizygus marmoreus* (19C)	0.084 ± 0.012	0.063 ± 0.013	0.738 ± 0.011	0.407 ± 0.050
*Hypsizygus marmoreus* (BY)	0.108 ± 0.026	0.087 ± 0.007	0.680 ± 0.007	0.477 ± 0.067
*Lentinula edodes*	0.161 ± 0.028	0.005 ± 0.001	1.073 ± 0.019	0.883 ± 0.014
*Agrocybe cylindracea*	0.232 ± 0.016	0.112 ± 0.003	0.997 ± 0.014	1.022 ± 0.077
*Flammulina velutipes*	0.304 ± 0.052	0.214 ± 0.026	1.097 ± 0.014	1.067 ± 0.033
*Pleurotus eryngii*	0.475 ± 0.034	0.145 ± 0.006	1.093 ± 0.009	0.673 ± 0.012
*Pleurotus pulmonarius*	0.514 ± 0.019	0.195 ± 0.017	1.240 ± 0.018	0.978 ± 0.084
*Pleurotus ostreatus*	0.519 ± 0.049	0.219 ± 0.022	1.330 ± 0.048	0.978 ± 0.034
*Volvaria volvacea*	0.763 ± 0.024	0.311 ± 0.027	2.620 ± 0.275	1.283 ± 0.126

### Analysis of glucose assimilation by nine edible fungi

**Figure 2:**
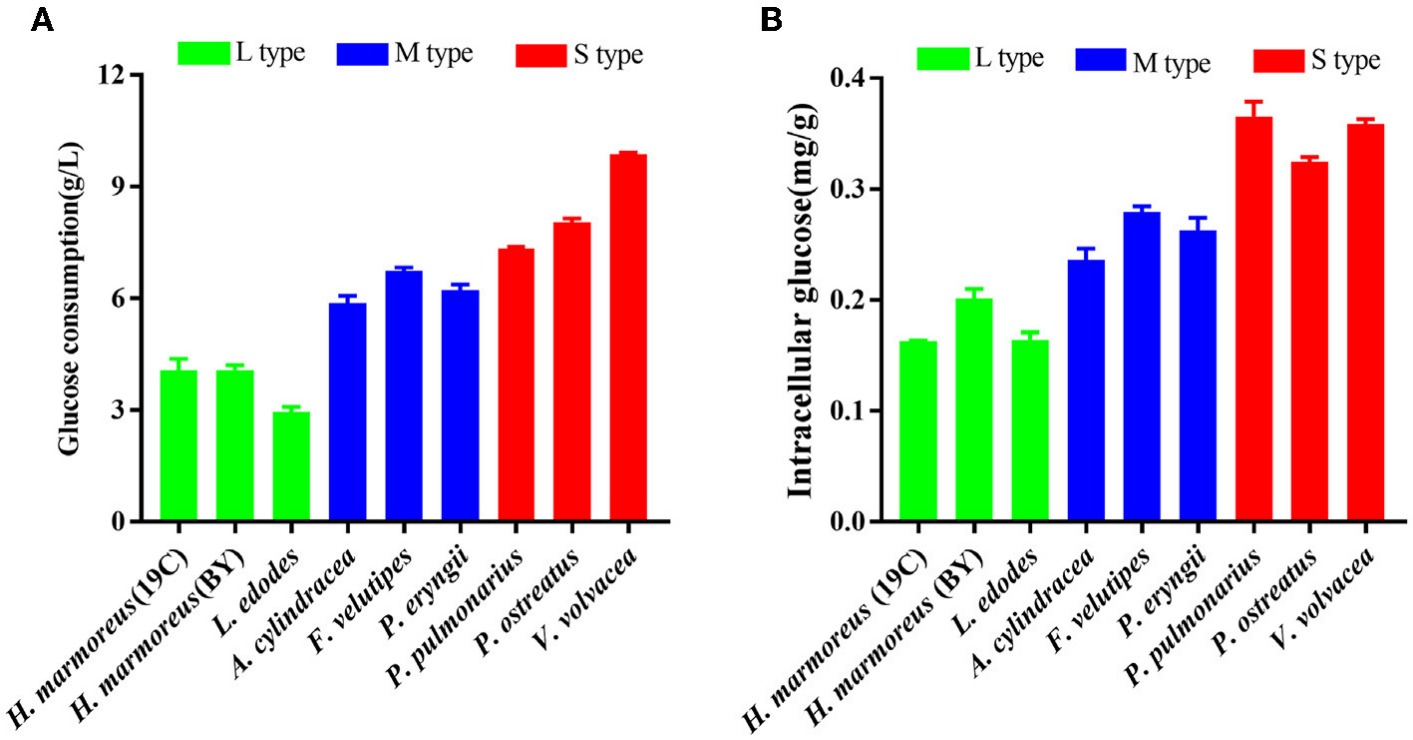
Assays of glucose assimilation rates on the liquid glucose medium by nine edible fungi. **(A)** Extracellular glucose consumption; **(B)** intracellular glucose concentration.

**Figure 3:**
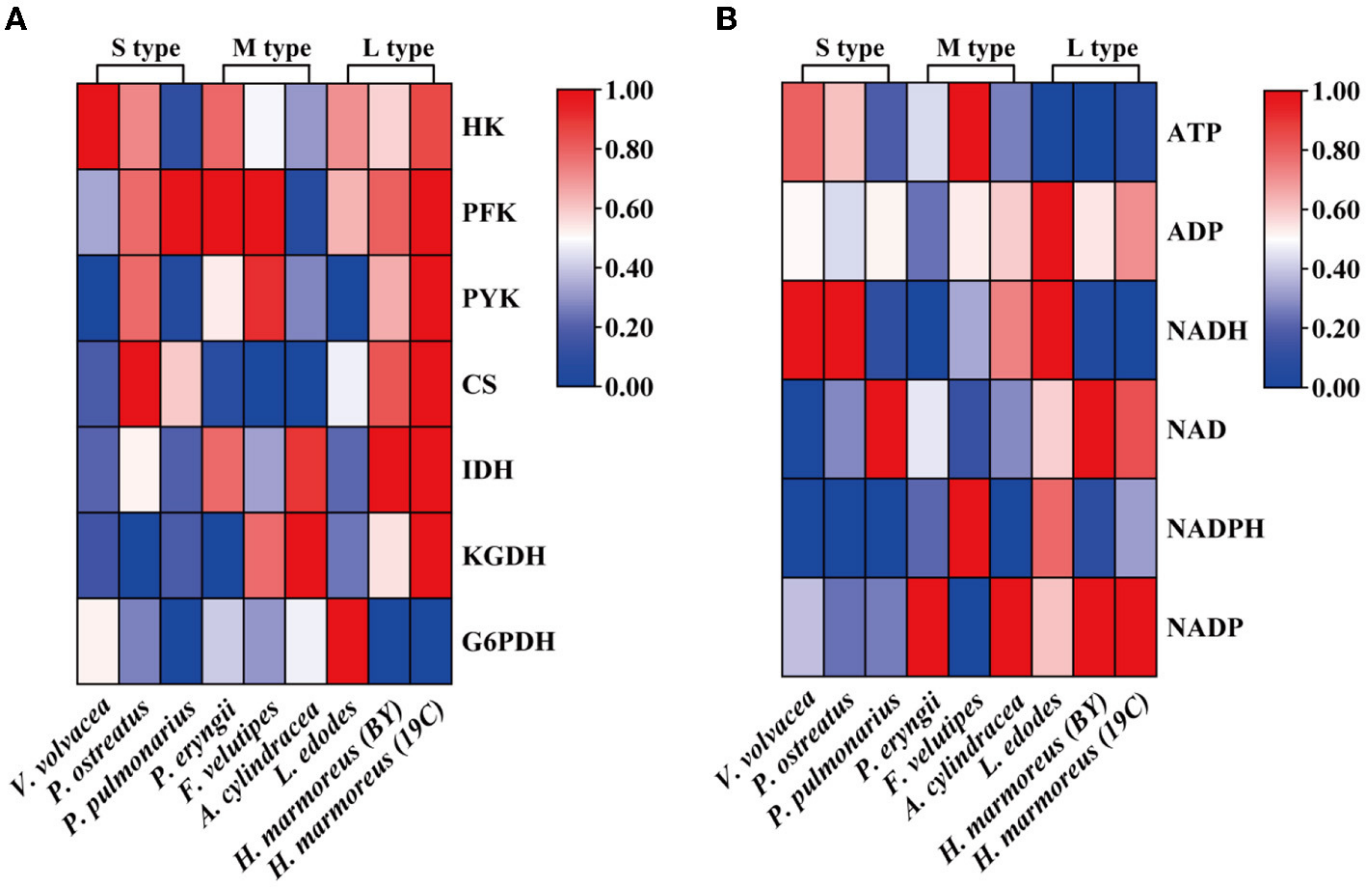
Heatmap analysis for the activities of seven key rate-limiting enzymes **(A)** and concentrations of six nucleosides **(B)** among nine edible fungi.

Glucose, as a direct carbon resource, was transported into cells by transporters and catabolized into raw materials and energy through glycometabolism for mycelia growth and development (Nogueira et al., 2018). Therefore, the levels of intracellular glucose assimilation could directly reflect the ability of *in vivo* nutrient and energy supplies. The glucose consumption in the liquid GM medium by nine edible fungi is given in Figure 2A. The results showed that extracellular glucose consumption rates by these nine strains appeared to be decreasing as the harvest period increased, and the L-type strains with the slowest glucose consumption rates could be observed. Similarly, intracellular glucose concentrations among the nine strains also exhibited decreasing trends in the order of S-, M-, and L-type strains (Figure 2B). The relatively low concentrations of intracellular glucose as a substrate for L-type strains might influence carbon flux distribution into the branches of CCM by altering the reaction velocities and/or expressions of the related enzymes. As reported in a previous study, glucose supplemented in the medium could significantly alter the transcriptional levels of over 5% of genes in the genome of *Streptococcus pneumoniae* (Neves et al., 2002; Paixão et al., 2015). To reveal the reasons resulting in significant differences in glucose assimilation rates, the activities of seven key rate-limiting enzymes involved in EMP (hexokinase, phosphofructokinase, and pyruvate kinase), PPP (glucose-6-phosphate dehydrogenase), and the TCA cycle (citrate synthase, isocitrate dehydrogenase, and 2-ketoglutarate dehydrogenase) of CCM were assayed for the nine edible fungi cultured in the GM medium. The enzyme activities of nine edible fungi were compared and are given in Figure 3A. The heatmap analysis indicated that these key enzyme activities had no noticeable distribution pattern among nine edible fungi with different glucose assimilation rates. However, the L-type strains of *H. marmoreus* (19C and BY) showed a similar distribution with higher enzyme activities in the EMP/TCA cycle and lower enzyme activity of G6PDH in PPP, suggesting that an imbalanced distribution of enzyme activities in the three branches of CCM might result in a low glucose assimilation rate as well as the slower growth rate of *H. marmoreus*. Interestedly, the *L. edodes* belonging to the L-type strain exhibited higher G6PDH activity in PPP and lower activity of pyruvate kinase (PYK) in EMP. In contrast, the seven key enzyme activities among the other six edible fungi appeared to have a relatively balanced distribution. The above results showed that the imbalanced distribution of enzyme activities in dynamic flux control mode in the three branches of CCM might influence raw materials and energy supply in individual pathways and thus delay the growth and development of the L-type strains. Xiong et al. (2021) found that an imbalanced flux distribution in CCM limited the high production of L-tryptophan in *E. coli*. The strategy of flux redistribution in the CCM of *E. coli* was developed to improve phosphoenolpyruvate and erythrose-4-phosphate pools, thus leading to efficient L-tryptophan synthesis. Furthermore, the concentrations of six nucleosides, including ADP, NAD, NADP, ATP, NADH, and NADPH among the nine edible fungi cultured in the GM medium, were assayed and compared by heatmap analysis. As shown in Figure 3B, the L-type edible fungi of *H. marmoreus* (19C), *H. marmoreus* (BY), and *L. edodes* exhibited high levels of ADP and low levels of ATP, indicating that these three strains lacked adequate energy supplies partially due to their slow glucose assimilation rates. As reported by Yang et al. (2021), higher biomass with faster glucose consumption and more ATP generation could be achieved through Tween 80 supplementation during the culture process of *Ganoderma lucidum*. In addition, the high concentration of oxidized coenzymes and low concentration of reduced coenzymes for *H. marmoreus* (19C) and *H. marmoreus* (BY) could be observed, which was consistent with weaker enzyme activities in PPP and thus led to a decreased supply of reducing power in the process of biomacromolecular synthesis and cell proliferation. For another L-type strain of *L. edodes*, a relatively higher concentration of reduced coenzymes than *H. marmoreus* could be detected, partially due to higher G6PDH activity in PPP (Figure 3A). Combined with the enzymes PYK in EMP and CS, IDH, and KGDH in TCA with low enzyme activities (Figure 3A), a slow growth rate of *L. edodes* might be attributed to a low level of TCA, as a high level of ADP and a low level of ATP could be observed (Figure 3B). Based on the above results, we assumed that the imbalanced distribution of CCM, which presented high metabolic levels of the EMP and TCA cycles and a low PPP metabolic level, might contribute to a slow mycelial growth rate and a long harvest period for *H. marmoreus* (19C and BY) to a certain extent.

### Validation of a low PPP metabolic level in *H. marmoreus*

**Figure 4:**
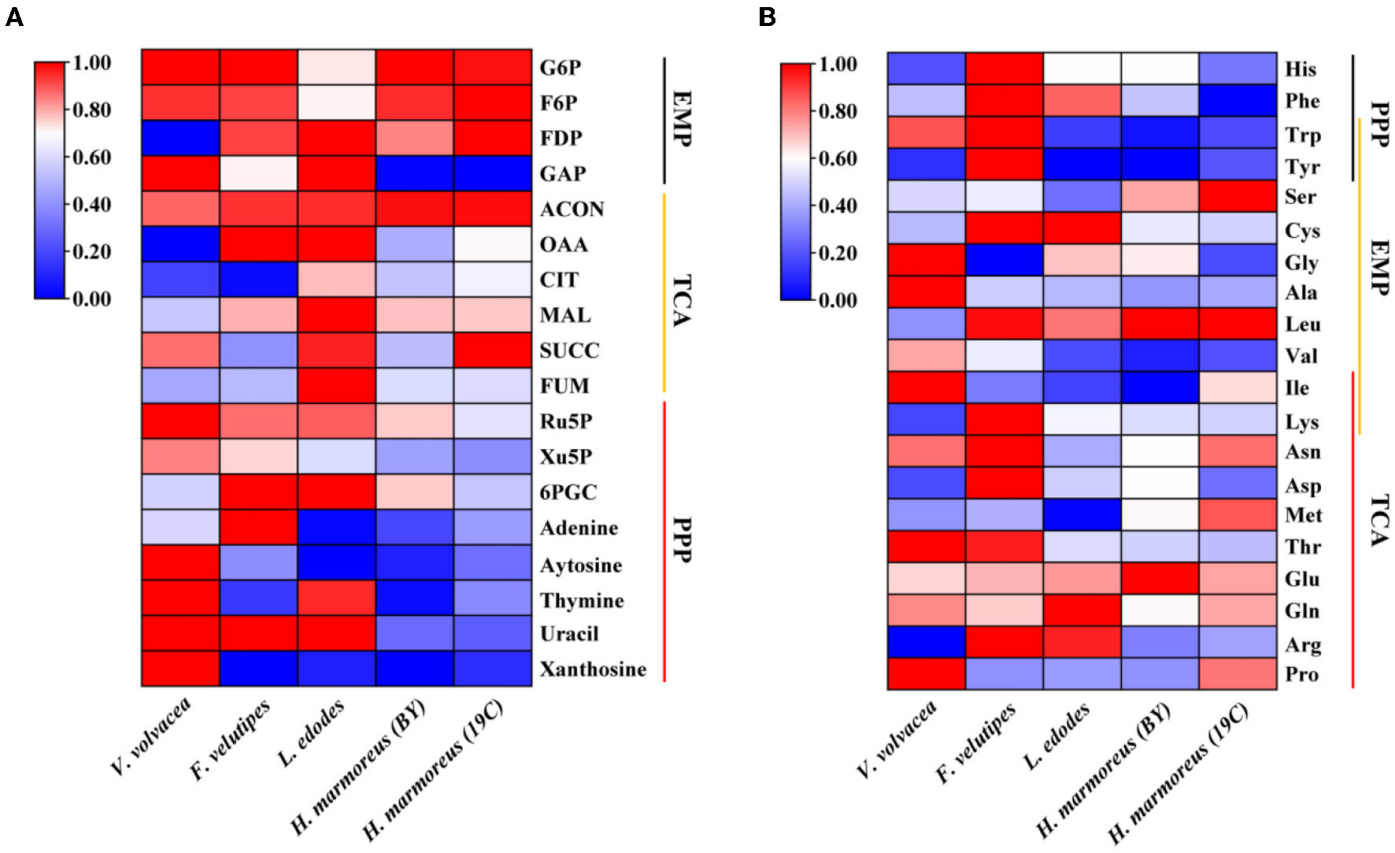
Heatmap analysis of intermediate metabolites, nucleotides, and amino acids concentration in *Hypsizygus marmoreus* and the control strains. **(A)** Intermediate metabolites and nucleotides; **(B)** amino acids.

**Figure 5:**
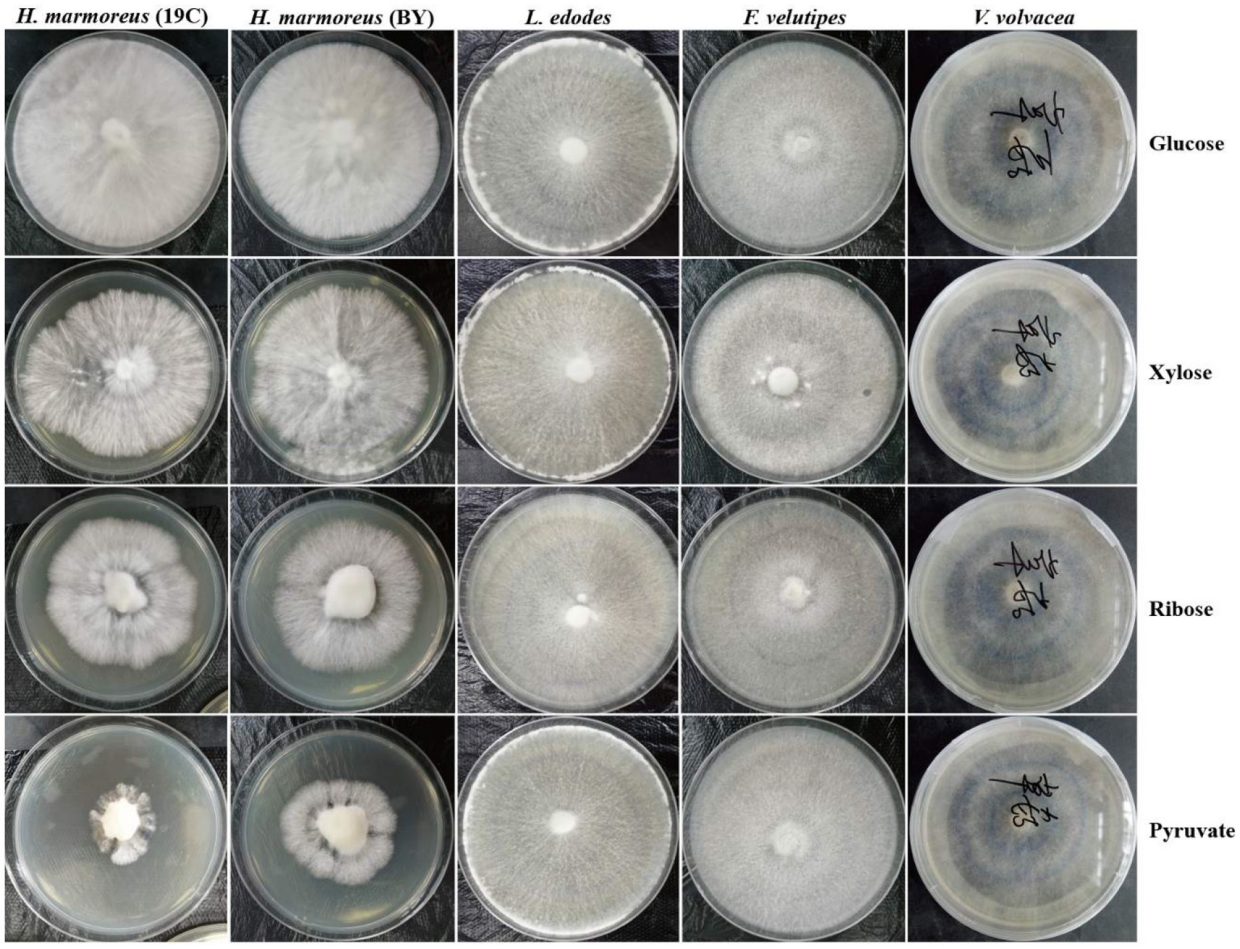
Mycelial growth of *Hypsizygus marmoreus* and the control strains on various carbon sources.

To prove our above inference, three methods, including metabolomics, adaptation of various carbon sources, and chemical interference, were employed to validate the slow mycelial growth rate and attribute it partially to the imbalanced distribution of the CCM in *H. marmoreus*. For metabolomics analysis, the data of intermediate metabolites, nucleotides, and amino acids were collected and compared by heatmap analysis. As shown in Figure 4A, the accumulation of intermediate metabolites and nucleotides involved in the CCM exhibited similar results between *H. marmoreus* (19C) and *H. marmoreus* (BY), indicating that the two strains possessed the same metabolic levels during the growth process. Except for glyceraldehyde-3-phosphate (GAP), the concentrations of intermediate metabolites from the EMP and TCA cycles in *H. marmoreus* showed no evident difference when compared to control strains, including *L. edodes, F. velutipes*, and *V. volvacea*. However, both the M- and L-type strains showed insufficient accumulation of intermediate metabolites and nucleotides derived from the PPP in comparison to the S-type strain of *V. volvacea*. In particular, the nucleotide pools with low contents in *H. marmoreus* (19C) and *H. marmoreus* (BY) could be observed, suggesting that the carbon flux into the PPP for the two strains was significantly weak (Figure 4A). Duan et al. (2010) found that alteration of the carbon flux into the PPP through the overexpression of G6PDH in *Bacillus subtilis* could efficiently increase the availability of ribulose-5-phosphate and result in a higher riboflavin yield. However, the contents of each amino acid derived from the CCM were assayed among the tested strains and are depicted in Figure 4B. The results revealed that the amino acid contents appeared to have a different distribution, and only the M-type strain of *F. velutipes* exhibited high accumulation for each amino acid. The L-type strain of *L. edodes* showed a significant imbalance of amino acid accumulation, and the amino acids Trp, Tyr, Val, Ile, and Met, with low concentrations, could be observed in *L. edodes*. For *H. marmoreus* (19C) and *H. marmoreus* (BY), a relatively low concentration of Phe, Trp, and Tyr derived from PPP could be detected through other amino acids exhibiting different distributions (Figure 4B). Metabonomics analysis indicated that the low level of intermediate metabolites, especially nucleotides, derived from the PPP was a major factor resulting in the slow mycelial growth rates of *H. marmoreus*. Diehl et al. (2022) also showed that nucleotide imbalances decoupled cell growth from cell proliferation. Subsequently, four carbon sources, including glucose, xylose, ribose, and pyruvate, were used to cultivate *H. marmoreus* to investigate its adaptability to carbon sources, and the strains of *L. edodes, F. velutipes*, and *V. volvacea* were used as controls. Different carbon sources as initial nutrients could be utilized and entered into carbon metabolic pathways to provide raw materials and energies via different approaches in edible fungi, which somewhat reflected metabolic levels of EMP, TCA cycle, and PPP in the CCM (Supplementary Figure 3). The results showed that four carbon sources had a minor effect on the mycelial growth of all the tested strains except *H. marmoreus* (Supplementary Table 2 and Figure 5). *Hypsizygus marmoreus* could readily utilize glucose as a carbon source and thus result in a normal growth rate. While xylose and ribose as carbon sources showed reduced 30 and 46% mycelial growth rates compared to those of *H. marmoreus* by glucose. A possible explanation was that the low metabolic level of the PPP in *H. marmoreus* influenced the pentose assimilation rate and thus reduced the supply of intermediate metabolites and reduced power. In *Yarrowia lipolytica*, the expression levels of xylose transporters, the xylose-utilization pathway, and downstream PPP were optimized to improve the pentose uptake rates and maximize the metabolic flux toward the final products (Ryu and Trinh, 2018; Sun et al., 2021). Pyruvate was not a carbon source for the mycelial growth of *H. marmoreus* (Supplementary Table 2 and Figure 5), which might be attributed to the gluconeogenesis disorder and subsequent insufficient intermediate metabolite supply mediated by PPP. Finally, seven kinds of interferent agents were used to explore the roles of the EMP, PPP, and TCA cycles for mycelial growth rates of *H. marmoreus* and the control strains by interfering with the CCM. The mechanisms of all the interferent agents are given in Supplementary Figure 4. The results showed that succinate and malonate as activators and inhibitors, respectively, for the TCA cycle had no effects on the mycelial growth of all the tested strains except for the S-type strain of *V. volvacea*, which was inhibited by malonate (Table 3). Similarly, no noticeable effects were observed on mycelial growth by DCA as an inhibitor of EMP among all the tested strains. These results indicated that the metabolic pathways of EMP and TCA cycling among all the tested strains were rigid during the growth process. In contrast, the mycelial growth of *H. marmoreus* could be significantly influenced by chemical inhibitors and activators of PPP. 6AN and DEHA as inhibitors of G6PDH limiting carbon flux into PPP significantly reduced the mycelial growth rates of both *H. marmoreus* (19C) and *H. marmoreus* (BY), while the mycelia of both *H. marmoreus* appeared thicker with a slight increase in growth rate by SAHA and VA as activators of G6PDH in PPP (Table 3 and Supplementary Figure 5). However, the four activators and inhibitors involved in PPP had no evident effect on the mycelial growth rates of the control strains. These results showed that the PPP of CCM in *H. marmoreus* appeared to have good flexibility, and its carbon flux might be readily adjusted in *H. marmoreus* by environmental factors or molecular manipulation. Together with the above results of metabolomics, adaptation of various carbon sources, and chemical interference, a low metabolic level of the PPP and an imbalanced distribution of the CCM in *H. marmoreus* were two of the major factors that resulted in a slow carton source assimilation rate and a low mycelial growth rate, which provided new insights for breeding *H. marmoreus*.

**Table 3:** The effects of seven interferent agents on mycelial growth rates of *Hypsizygus marmoreus* and the control strains.

**Interferent agents**	**Average growth rate (cm/day)**				
	***Hypsizygus marmoreus*** **(19C)**	***Hypsizygus marmoreus*** **(BY)**	***Lentinula edodes***	***Flammulina velutipes***	***Volvaria volvacea***
CK	0.469 ± 0.005	0.511 ± 0.005	0.489 ± 0.010	0.530 ± 0.028	0.878 ± 0.025
6AN	0.040 ± 0.000	0.040 ± 0.000	0.478 ± 0.010	0.526 ± 0.017	0.928 ± 0.025
DEHA	0.136 ± 0.010	0.302 ± 0.014	0.494 ± 0.005	0.533 ± 0.029	0.733 ± 0.011
SAHA	0.542 ± 0.008	0.522 ± 0.013	0.491 ± 0.008	0.526 ± 0.023	0.867 ± 0.017
VA	0.511 ± 0.005	0.514 ± 0.013	0.142 ± 0.010	0.515 ± 0.042	0.917 ± 0.017
DCA	0.478 ± 0.012	0.510± 0.009	0.492 ± 0.120	0.518 ± 0.009	0.891 ± 0.021
Malonate	0.454 ± 0.007	0.509 ± 0.004	0.483 ± 0.007	0.522 ± 0.014	0.752 ± 0.028
Succinate	0.471 ± 0.015	0.512 ± 0.010	0.491 ± 0.011	0.536 ± 0.016	0.865 ± 0.013

### Enhanced g6pdh expression level for improving the growth and development of *H. marmoreus*

**Figure 6:**
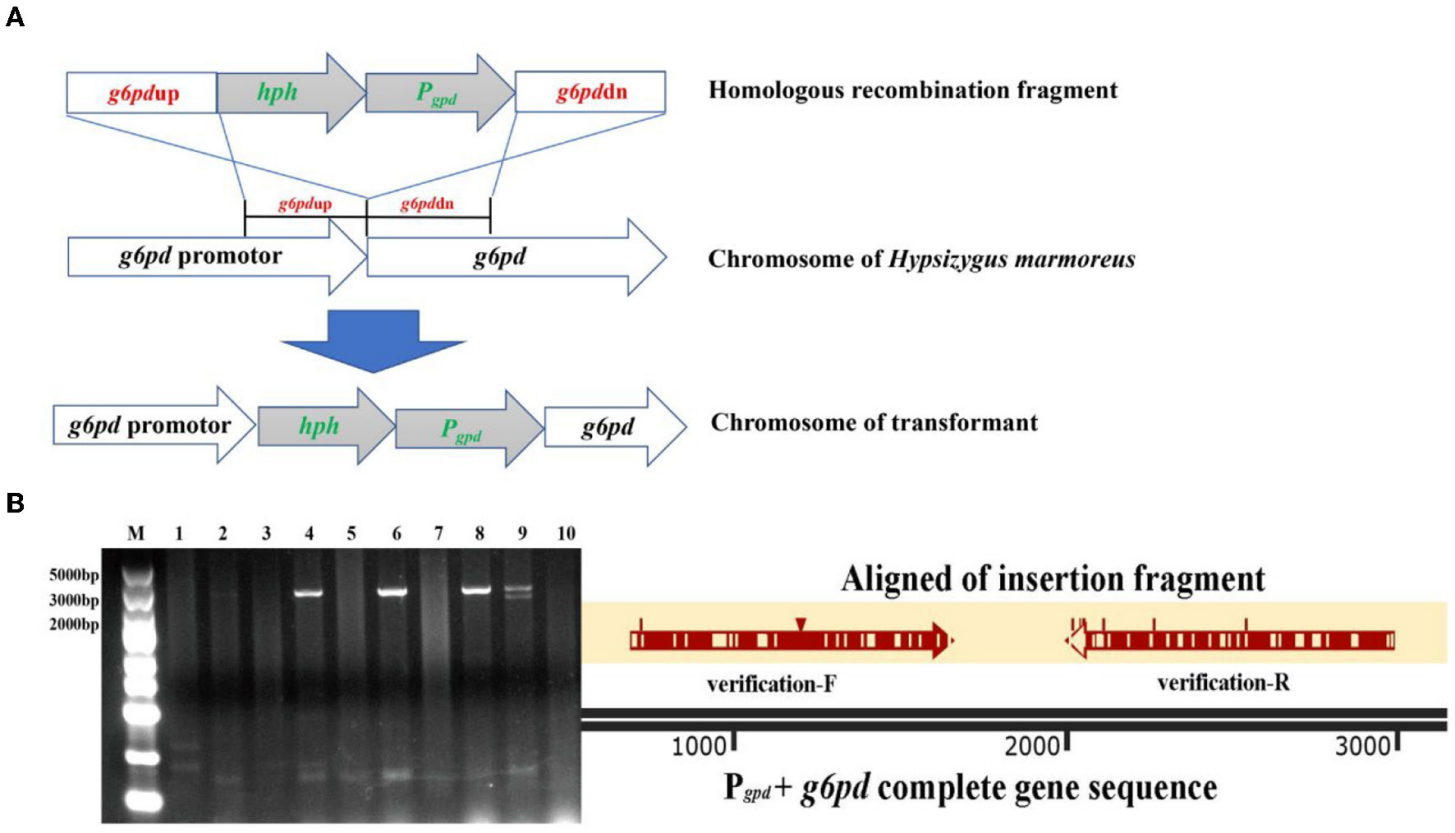
The transformants with improved G6PDH expression level via GPD promoter instead of its native promoter in *Hypsizygus marmoreus* (19C) and *H. marmoreus* (BY). **(A)** The outline of homologous recombination; **(B)** validation and alignment of insertion fragment (Lane M: DNA Marker; Lane 1: *H. marmoreus* 19C; Lane 2–6: transformants of 19C-1, 19C-4, 19C-6, 19C-17, 19C−21; Lane 7: *H. marmoreus* BY; and Lane 8–10: transformants of BY-1, BY-2, BY-5).

**Figure 7:**
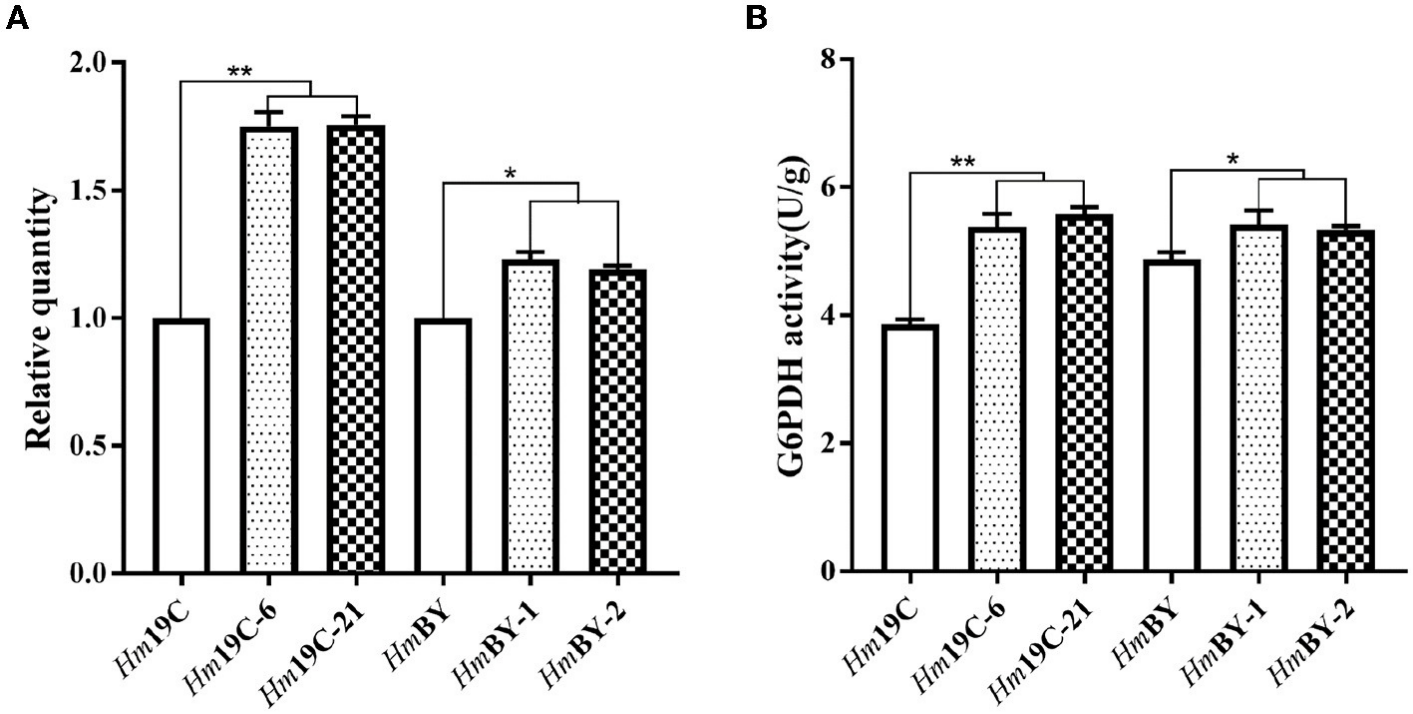
Quantitation of the expression levels for the *g6pd* gene **(A)** and G6PDH activity assays **(B)** in four transformants and wild-type strains. *Represented significant difference, *P* < 0.05; **represented extremely significant difference, *P* < 0.01.

Glucose 6-phosphate dehydrogenase (G6PDH), the first and key rate-limiting enzyme of PPP, controls the carbon flux, supplying raw materials and reducing power for biomacromolecule biosynthesis (Garcia et al., 2021). The low metabolic level of the PPP in *H. marmoreus* was one of the key factors that resulted in a slow mycelial growth rate. Four inhibitors or activators (6AN, DEHA, SAHA, and VA) regulating the expression level and enzyme activity of G6PDH in *H. marmoreus* could adjust the carbon flux into PPP and thus increase or decrease mycelial growth rate. Therefore, we attempted to increase the expression level of the gene *g6pd* by using a GPD promoter (P_*gpd*_) instead of its native promoter in *H. marmoreus*, as shown in Figure 6. The transformants were screened on resistant plates with hygromycin B and verified by amplifying insertion fragments and commercial sequencing. Ultimately, four transformants designated as *Hm*19C-6, *Hm*19C-21, *Hm*BY-1, and *Hm*BY-2 were achieved and used to determine their expression level by qRT-PCR and G6PDH enzyme activities. The results indicated that the expression levels of the *g6pd* gene in transformant *Hm*19C-6 and *Hm*19C-21 were enhanced by 75.0 and 75.5% when compared with those of wild type (Figure 7A), and the corresponding G6PDH activity with an increase of 39.4% and 44.3% were achieved (Figure 7B). However, two transformants of *Hm*BY-1 and *Hm*BY-2 showed a small increase, with 23.0 and 19.0% for expression levels and 11.1 and 9.4% for enzyme activities, compared to wild-type ones (Figure 7). These results showed that the GPD promoter, instead of the native promoter of the *g6pd* gene, could result in a higher expression level and enzyme activity of G6PDH, which could be used to evaluate the effect of increased G6PDH expression level from PPP on mycelial growth of *H. marmoreus*.

**Figure 8:**
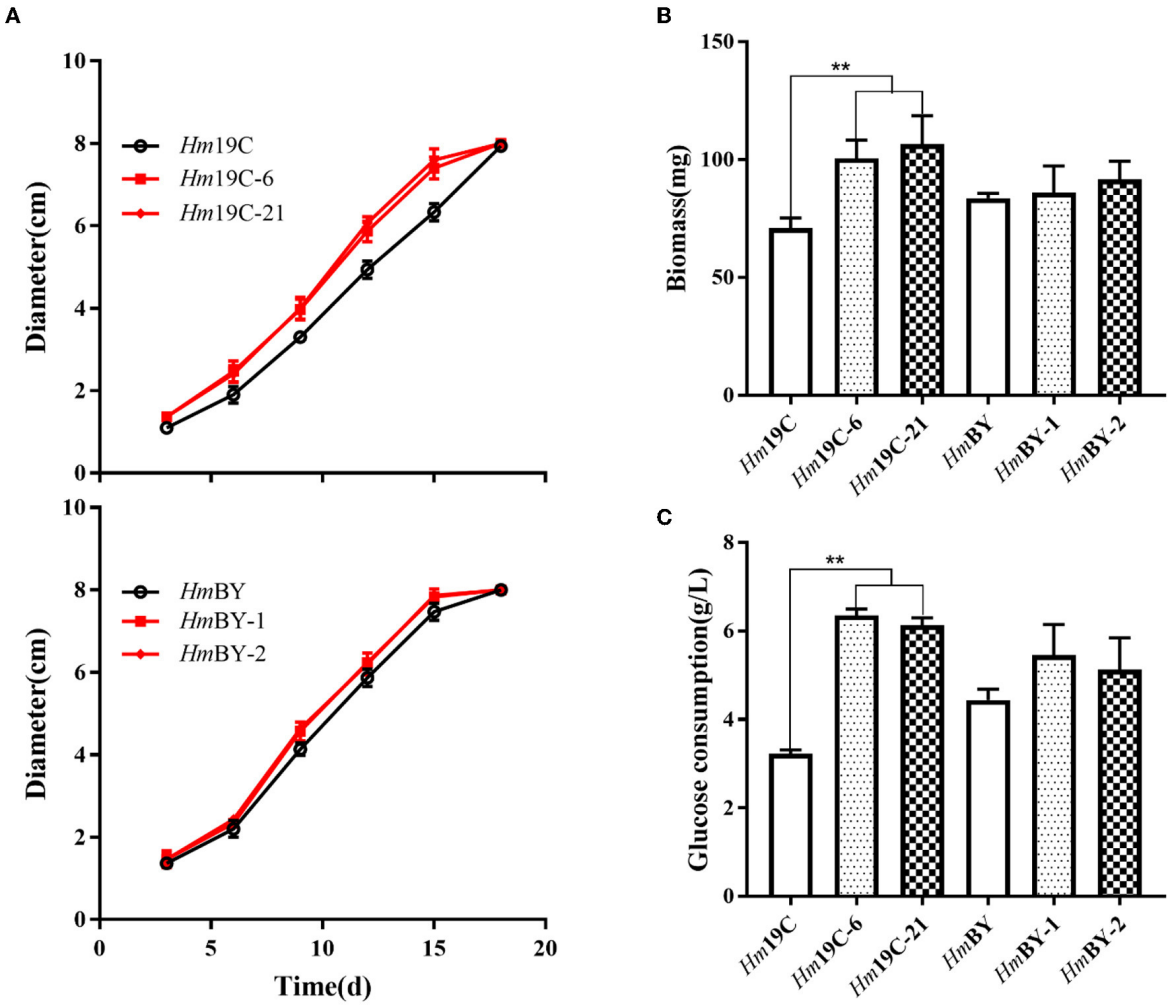
Assays of mycelial growth rates **(A)**, biomass **(B)**, and glucose consumption rates **(C)** by four transformants and wild-type strains by using glucose medium. **Represented significant difference, *P* < 0.01.

Subsequently, the mycelial growth rates, biomass, and glucose consumption rates on glucose medium were determined and compared between these transformants and the corresponding wild-type strains. As shown in Figure 8A, *Hm*19C-6 and *Hm*19C-21 showed faster mycelial growth rates with an improvement of ~26.8% when compared with the wild-type strain of *Hm*19C and a small increase in the mycelial growth rate by *Hm*BY-1 and *Hm*BY-2 could also be observed. Furthermore, the biomass and glucose consumption rates of *Hm*19C-6 and *Hm*19C-21 exhibited noticeable improvements in contrast with wild-type Hm19C. The biomass with an increase of 42.3 and 50.7%, and the glucose consumption rate with an almost one-fold improvement by *Hm*19C-6 and *Hm*19C-21 could be achieved (Figure 8B), while *Hm*BY-1 and *Hm*BY-2 only exhibited small increments in the biomass and glucose consumption rates (Figure 8C). The differences in mycelial growth rates, biomass, and glucose consumption rates were consistent with the expression levels and enzyme activities of G6PDH among the four transformants (Figure 7B). In previous studies, Sundara Sekar et al. (2017) found that the diversion of glycolytic flux from EMP to PPP in *E. coli* improved the co-production of hydrogen and ethanol by overexpressing G6PDH and 6-phosphogluconate dehdyrogenase (two major enzymes of the PPP) and disrupting phosphoglucose isomerase.

Similarly, overexpression of G6PDH in *Schizochytrium* sp. H016 promoted strain growth and glucose consumption and thus increased the availability of NADPH as well as docosahexaenoic acid production (Sundara Sekar et al., 2017; Feng et al., 2022). Therefore, increasing the G6PDH expression level in *H. marmoreus* could efficiently improve the glucose assimilation rate and promote mycelial growth and biomass production, which might contribute to increasing carbon flux into the PPP of *H. marmoreus*.

**Figure 9:**
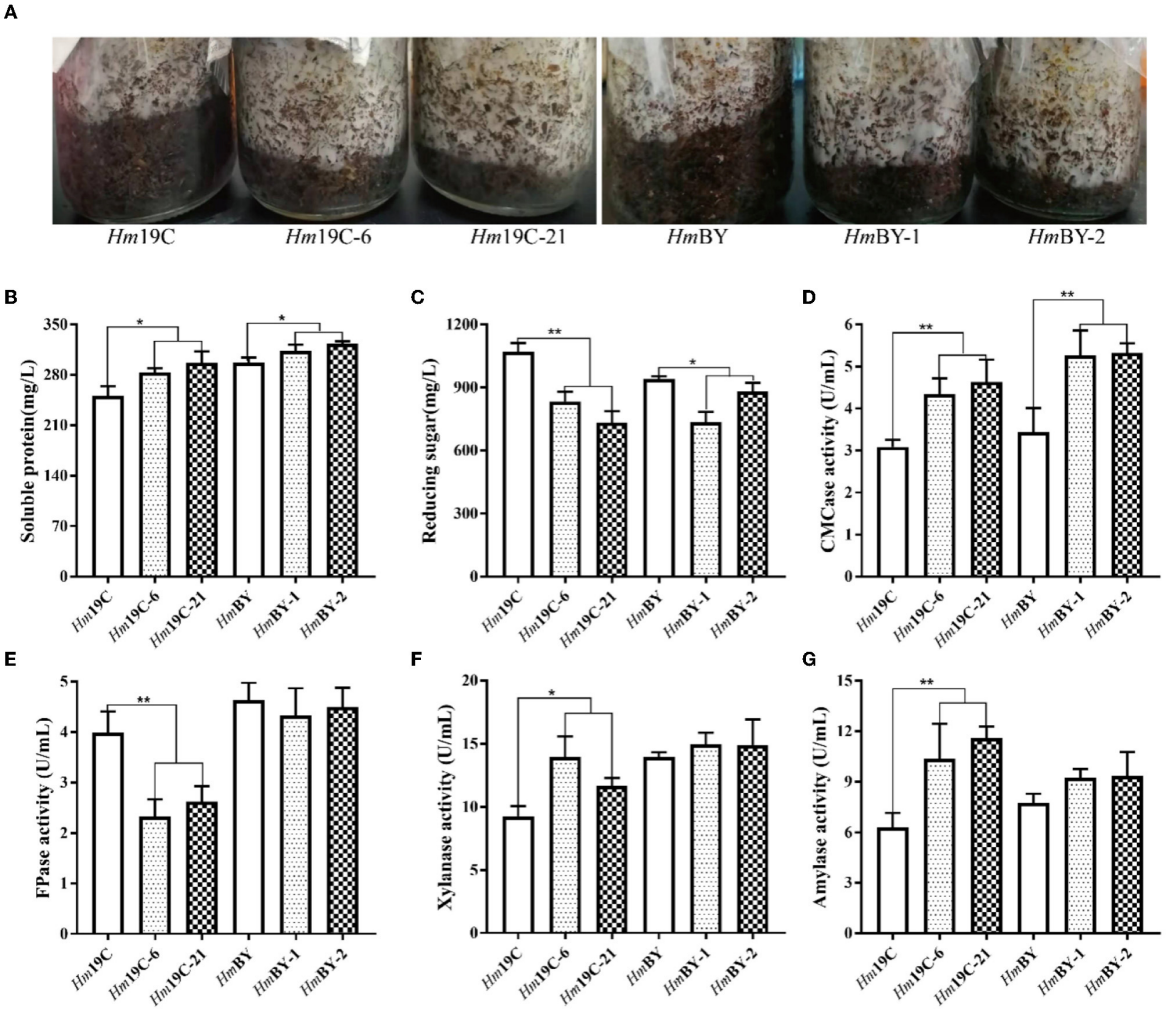
Assays of mycelial growth rates and extracellular metabolic parameters by four transformants and wild-type strains in bottle cultivation. **(A)** Mycelial growth rates; **(B)** Soluble protein secretion; **(C)** reducing sugar concentrations; **(D)** CMCase activities; **(E)** FPase activities; **(F)** Xylanase activities; **(G)** amylase activities. *Represented significant difference, *P* < 0.05; **represented extremely significant difference, *P* < 0.01.

Furthermore, these transformants and wild-type strains were inoculated into cultivation material mixtures to investigate the effects of the enhanced G6PDH expression level on mycelial growth rates and extracellular carbon metabolic levels. As shown in Figure 9A, four transformants exhibited faster mycelial growth rates in bottle substrate mixtures in comparison to wild-type strains, implying that improving the G6PDH expression level in *H. marmoreus* could promote mycelial growth when cultivation material mixtures were used as the medium. A reasonable explanation was that the increase in G6PDH expression level might result in changes in extracellular carbon metabolic levels among these transformants (Adnan et al., 2018). Therefore, four transformants and wild-type strains determined extracellular metabolic parameters, including soluble protein, reducing sugar, and the activities of CMCase, FPase, xylanase, and amylase. The increase in G6PDH expression level in *H. marmoreus* efficiently improved extracellular soluble protein secretion and accelerated the consumption of extracellular reducing sugar in bottle substrate mixtures by four transformants (Figures 9B, C). The increased secretion of soluble proteins resulted in activity changes in extracellular enzymes. The enzymes CMCase, xylanase, and amylase in bottle substrate mixtures by four transformants showed higher activities when compared with the wild-type strains (Figures 9D, F, G). However, the decreasing activity of FPase could be observed by these four transformants, especially *Hm*19C-6 and *Hm*19C-21 (Figure 9E). These results indicated that extracellular carbon metabolic levels could be regulated by adjusting intracellular carbon metabolic levels in *H. marmoreus*, though a detailed mechanism remained unknown.

**Figure 10:**
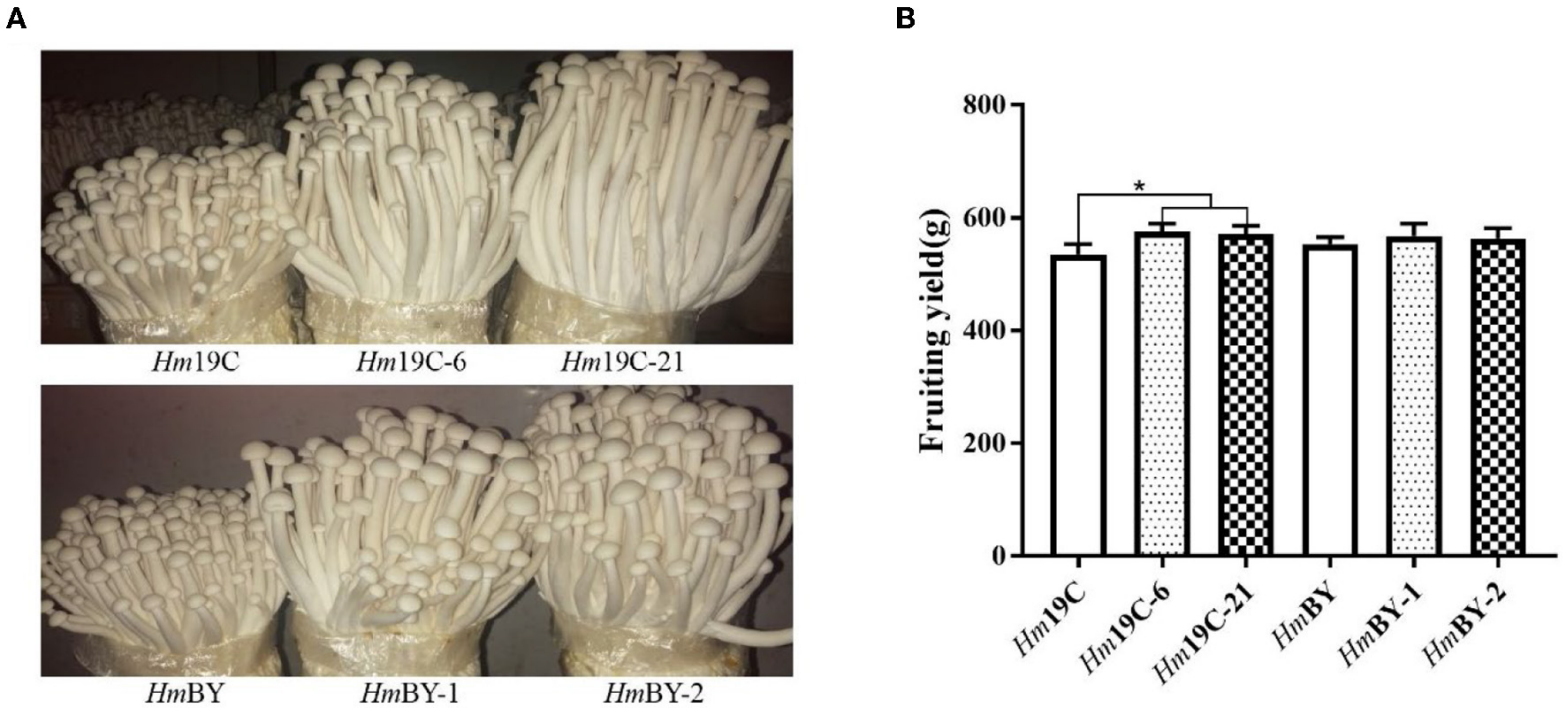
Fruiting tests of four transformants and the corresponding wild-type strains in bag cultivation. **(A)** The differences of the fruiting body. **(B)** The yield differences. *Represented significant difference, *P* < 0.05.

Finally, cultivation material mixtures were used to yield the fruiting body of *H. marmoreus* in bag cultivation and evaluated the effects of the enhanced G6PDH expression level on the fruiting body maturation period and fruiting body yield. Four transformants exhibited short fruiting body maturation periods ~4–5 days in advance, as shown in Figure 10A. The fruiting body yields of four transformants and wild-type strains were determined after maturation and harvest. The yields of *Hm*19C-6 and *Hm*19C-21 reached 574.8 g and 571.2 g per bag, respectively, with an increase of 7.4 and 7.3% in comparison to the wild-type strain *Hm*19C of 535.2 g per bag (Figure 10B). Although fruiting body yields with no significant differences among *Hm*BY-1, *Hm*BY-2, and *Hm*BY could be observed, only 2.7 and 1.8% increases in the yields of 568.2 and 563.4 g by *Hm*BY-1 and *Hm*BY-2 were achieved in contrast with 553.4 g of wild-type *Hm*BY (Figure 10B). These four transformants with different yields might be related to the expression levels and enzyme activities of G6PDH. Therefore, higher expression levels and enzyme activities of G6PDH achieved by using a strong promoter in *H. marmoreus* might lead to a higher yield of the fruiting body. Except for G6PDH, other attempts via adjusting the expression levels of sugar transporters and downstream PPP could be performed to redistribute the carbon flux of CCM, which might favor further improvement of the harvest period and fruiting body yield of *H. marmoreus*.

## Conclusions

This study revealed that the imbalanced distribution of CCM and low metabolic level of PPP in *H. marmoreus* resulted in a slow mycelial growth rate and a long harvest period. Further analysis showed that the PPP in *H. marmoreus* exhibited flexibility, and chemical activators and inhibitors readily altered its metabolic level. Carbon flux redistribution by enhancing the expression level of G6PDH in *H. marmoreus*, a rate-limiting enzyme of PPP, resulted in a faster mycelial growth rate, reduced maturation periods, and increased fruiting body yield. The results provided new insights for the breeding of edible fungi in the future.

## Data availability statement

The original contributions presented in the study are included in the article/Supplementary material, further inquiries can be directed to the corresponding authors.

## Author contributions

LZ and KH conceived the idea and designed the experiment. HL, LM, PL, and SL performed the experiment. HL and LM collected the data and the samples. HL and PL performed the data analysis. HL and LZ wrote the manuscript. SS provided technical instruction. HL, LM, PL, SL, SS, KH, and LZ reviewed the manuscript. All authors contributed to the article and approved its submitted version.
