# Supplementary material for: Analysis and modification of central carbon metabolism in Hypsizygus marmoreus for improving mycelial growth performance and fruiting body yield
Source: Front Microbiol. 2023 Jul 25;14:1233512. doi: 10.3389/fmicb.2023.1233512 (PMC10407233; doi:10.3389/fmicb.2023.1233512)
Supplement: Supplementary file 1 [file Data_Sheet_1.PDF]

Supplementary Material

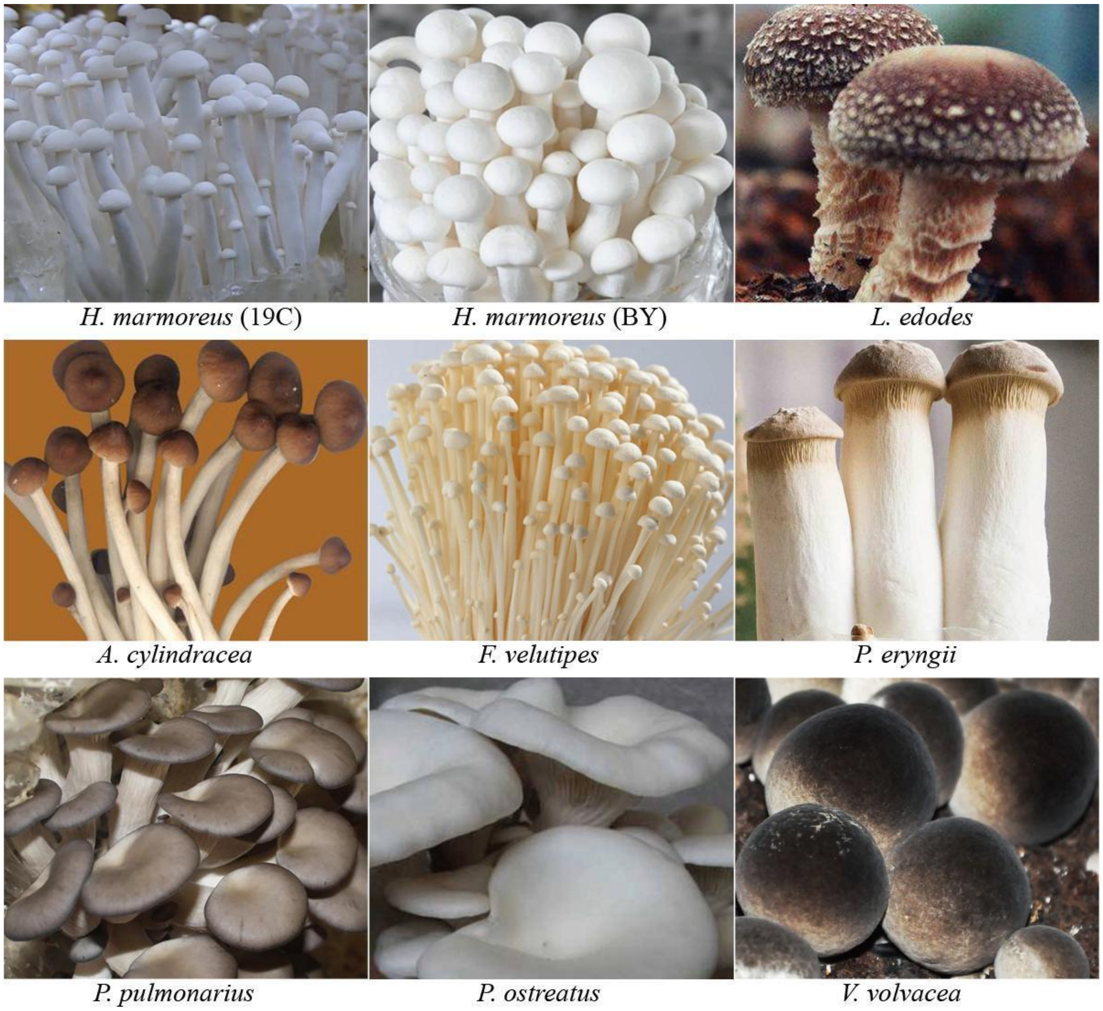

Supplementary Figure 1. Fruiting body morphology of 9 edible fungi collected from commercial mushroom farm in Fujian Province, China.

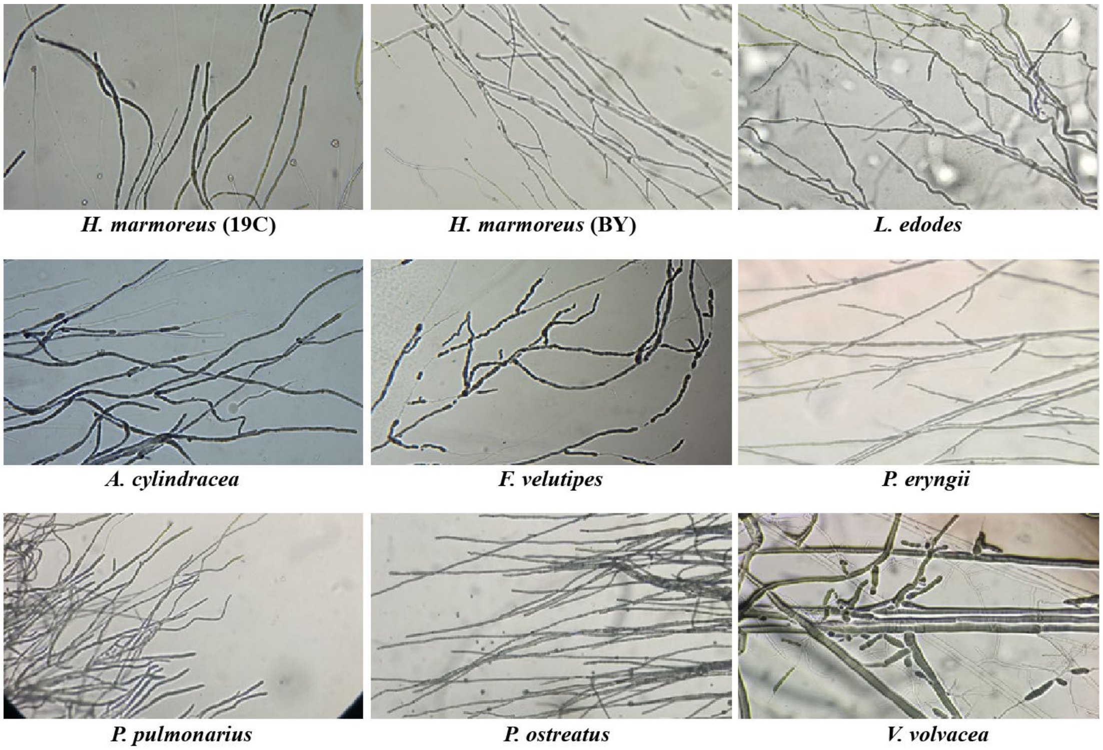

Supplementary Figure 2. Mycelial microscopic morphology of nine edible fungi

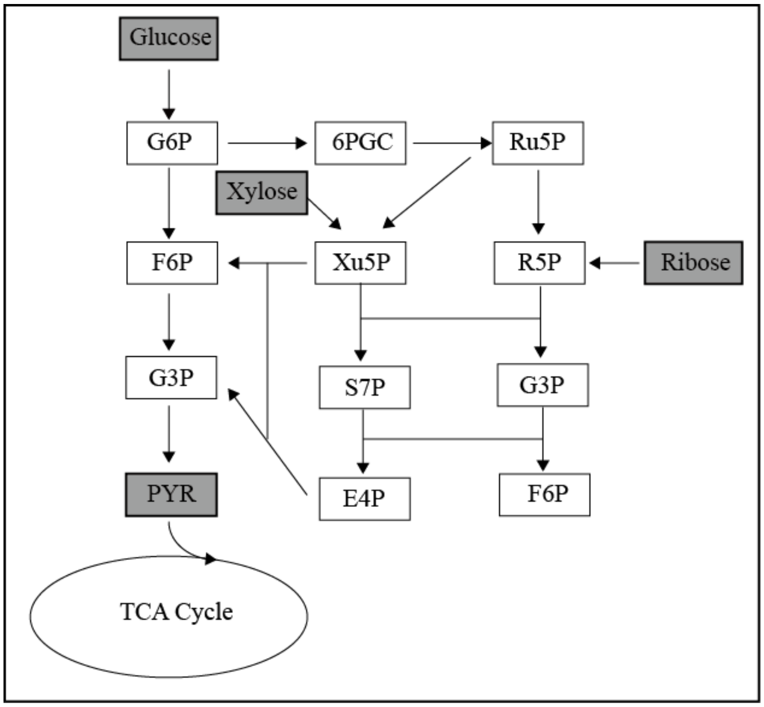

Supplementary Figure 3. Carbon metabolic pathway of four carbon sources

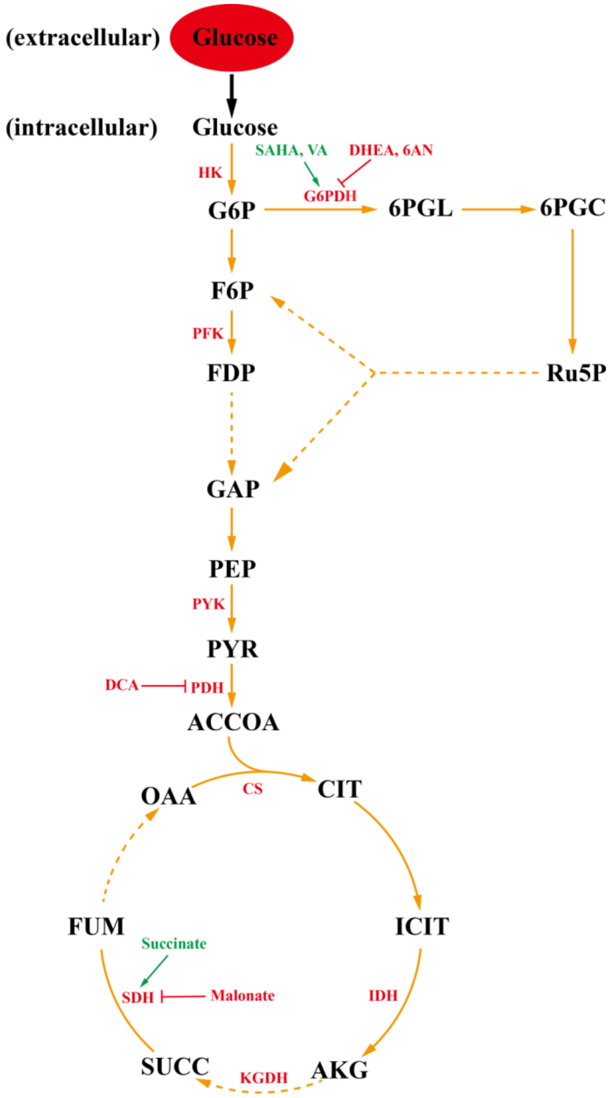

Supplementary Figure 4. The mechanisms of all the interferent agents for CCM

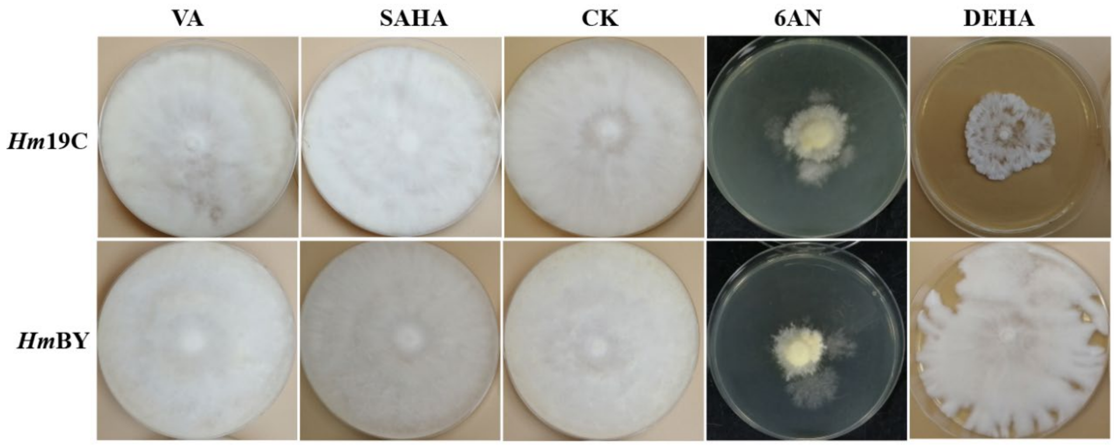

Supplementary Figure 5. The effects of activators and inhibitors on mycelial growth of *H. marmoreus*

Supplementary Table 1. Primers lists used in this study

Primers	Primers sequences (5’ -3’)	Used for
g6pdup-F	CGTCACAGGCTATTGGAATG	*g6pdup*
g6pdup-R	TCTGAACCGTGCTGCCAACA	
Hpt-F	TGTTGGCAGCACGGTTCAGA	*hph*
Hpt-R	CTATTCATATGTCGCTCGAGG	
Hmgpd-F	CCTCGAGCGACATATGAATAG	P_{gpd}
Hmgpd-R	GGTGAATGTGTGTTGTTTGGG	
g6pddn-F	CCAAACAACACACATTCACCA	*g6pddn*
g6pddn-R	TACCGCATAAGATGAGCCAC	
RepUC19-F	AAGCTTGGCGTAATCATGGTCATAGC	pUC19
RepUC19-R	GGATCCCCGGGTACCGAGCTCGAATT	
Hpt-F2	CTCGTGCTTTCAGCTTCGA	Screen
Hpt-R2	CTGTTATGCGGCCATTGTC	
gpdg6pd-F	TTTGGGTCTGCCAGGTTT	Screen
gpdg6pd-R	AGCCGTACTTGGTGATGAAA	
ITS2-F	CTTGGTCATTTAGAGGAAGTAA	qPCR primers for housekeeping genes
ITS2-R	GCTGCGTTCTTCATCGATGC	
g6pd-F	GGATACGCTCGCACGAAGAT	qPCR primers for *g6pd* gene
g6pd-R	GGAGGGAGGGCAAAGTAGAAA	

Supplementary Table 2. The effects of different carbon sources on mycelial growth rates of *H. marmoreus* and the control strains

Carbon sources	Average growth rate (cm/d)				
	H. marmoreus (19C)	*H. marmoreus* (BY)	*L. edodes*	*F. velutipes*	*V. volvacea*
Glucose	0.367 ± 0.012	0.376 ± 0.015	0.489 ± 0.001	0.541 ± 0.023	0.894 ± 0.025
Xylose	0.247 ± 0.007	0.262 ± 0.010	0.503 ± 0.017	0.57 ± 0.013	0.944 ± 0.025
Ribose	0.198 ± 0.014	0.211 ± 0.004	0.462 ± 0.010	0.514 ± 0.010	0.744 ± 0.011
Pyruvate	0.067 ± 0.007	0.109 ± 0.004	0.467 ± 0.007	0.494 ± 0.010	0.752 ± 0.017
